# *Helicobacter pylori*‐induced exosomal MET educates tumour‐associated macrophages to promote gastric cancer progression

**DOI:** 10.1111/jcmm.13847

**Published:** 2018-08-30

**Authors:** Ying Che, Biao Geng, Yue Xu, Xin Miao, Ling Chen, Xianmin Mu, Jinshun Pan, Chen Zhang, Ting Zhao, Chao Wang, Xiang Li, Hao Wen, Zheng Liu, Qiang You

**Affiliations:** ^1^ Medical Center for Digestive Diseases Second Affiliated Hospital Nanjing Medical University Nanjing Jiangsu China; ^2^ Department of Biotherapy Second Affiliated Hospital Nanjing Medical University Nanjing Jiangsu China; ^3^ Department of Surgery Second Affiliated Hospital Nanjing Medical University Nanjing Jiangsu China; ^4^ Key Laboratory for Aging & Disease Nanjing Medical University Nanjing Jiangsu China

**Keywords:** exosome, gastric cancer, *Helicobacter pylori*, MET, tumour‐associated macrophage

## Abstract

*Helicobacter pylori (H. pylori)* infection triggers chronic inflammation that has been associated with gastric cancer (GC). Exosomes are small extracellular vesicles that have become the key mediators of intercellular communication. In this study, we investigated exosome‐mediated communication between *H. pylori*‐infected GC cells and macrophages, focusing on the transfer of activated mesenchymal‐epithelial transition factor (MET). We observed a significant decrease in MET protein expression in GC cells after infection with *H. pylori*, whereas MET mRNA levels remained unchanged. Intriguingly, MET expression, specifically the phosphorylated active form, was increased in exosomes released from *H. pylori*‐infected GC cells. Confocal microscopy and Western blotting analyses showed that these exosomes containing MET were delivered to and internalized by macrophages. Indeed, in human GC tissues positive for *H. pylori*, we also observed that activated MET was highly expressed in tumour‐infiltrating macrophages. After internalization, exosomal MET then appeared to educate the macrophages towards a pro‐tumorigenesis phenotype. This included exosomal MET‐mediated stimulation of proinflammatory cytokine secretion IL‐1β, which subsequently promoted tumour growth and progression in vitro and in vivo. Taken together, these data were the first to demonstrate *H. pylori* infection‐induced upregulation of activated MET in exosomes and the pro‐tumorigenic effect on tumour‐associated macrophages.

## 1 INTRODUCTION

Gastric cancer (GC) is the fourth most common malignancy and the third leading cause of cancer mortality worldwide.^1^ *Helicobacter pylori* has been considered a major risk factor for the development of GC^2^ and affects almost 80% of GC patients.^3^ Infection with *H. pylori* induces an inflammatory response and aberrant activation of immune cells, which contributed to GC pathogenesis.^4^

The cell‐surface receptor tyrosine kinase mesenchymal‐epithelial transition factor (MET) plays a critical role in tumour development, invasion and angiogenesis in solid tumour malignancies.^5^ MET is activated via phosphorylation at Tyr1234/1235,^6^ and this activated form functions as a key scaffolding protein in multiple intracellular signalling pathways.^7^ Notably, the *H. pylori* effector protein CagA intracellularly targets the MET receptor, resulting in robust MET phosphorylation, activation of downstream cellular processes and a forceful motogenic response (cell scattering).^8^ Intriguingly, MET can be packed in exosomes secreted from melanoma cells and subsequently educated bone marrow progenitor cells towards a pro‐metastatic phenotype.^9^

Exosomes are 40‐150 nm bilayer membrane vesicles that have recently been recognized as important mediators of intercellular communication, as they contain a wide range of functional lipids, proteins, RNA and DNA that can be transferred to a recipient cell via fusion of the exosome with the target cell membrane.^10^, ^11^ It has been shown that EGFR in the exosomes secreted by GC cells regulates liver microenvironment and facilitates the metastasis of GC cells to liver.^12^ These tumour‐derived exosomes (TEXs) are also known to be involved in the recruitment of neutrophils and the activation of macrophages,^13^ and influence the antitumour activity of immune cells through transferring suppressive or activating molecular signals.^14^

In this study, we investigated the exosome‐mediated communication between *H. pylori* ‐infected GC cells and macrophage, focusing on the transfer of activated MET. Furthermore, the downstream effects of this communication on proinflammatory factors IL‐1β were also evaluated. To our knowledge, this is the first time the mechanism by which *H. pylori* infection reshapes the immune microenvironment and contributes to the progression of GC has been evaluated.

## 2 MATERIALS AND METHODS

### 2.1 Cell culture and co‐culture with *H. pylori*

The gastric cancer cell lines AGS, MGC‐803 and SGC‐7901 were purchased from the cell bank of the Chinese Academy of Sciences (Shanghai, China) and cultured in Dulbecco's Modified Eagle Medium (DMEM; Invitrogen, Carlsbad, CA, USA) supplemented with 10% foetal bovine serum (FBS; Gibco, Grand Island, NY, USA) and 1% penicillin/streptomycin (Thermo Scientific, Waltham, MA, USA). Cells were cultured at 37°C under a humidified atmosphere including 5% CO_2_. *H. pylori* strains were cultured on blood agar plates containing 5% horse serum at 37°C in a microaerobic atmosphere and were harvested via centrifugation. The densities of bacteria were determined by measuring the optical density (OD) at 660 nm. 1OD_660_ = 1 × 10^8^ colony‐forming units (CFU)/mL. GC cells were co‐cultured with *H. pylori* at an optimal multiplicity of infection (MOI) of 100 for different time‐points as determined previously.^15^

### 2.2 Differentiation of THP‐1 monocytes into macrophages

The human monocytic cell line THP‐1 was purchased from the cell bank of the Chinese Academy of Sciences (Shanghai, China). THP‐1 cells were cultured in Roswell Park Memorial Institute (RPMI) 1640 medium (Invitrogen) supplemented with 10% heat‐inactivated FBS (Gibco), 10 mmol/L HEPES, 2 mmol/L glutamine, 100 U/L penicillin and 100 mg/mL streptomycin and maintained at 37°C under 5% CO_2_. Further, THP‐1 cells were differentiated into macrophages by incubation with 5 ng/mL phorbol 12‐myristate 13‐acetate (PMA; Sigma, St. Louis, MO, USA) for 48 hours.^16^

### 2.3 Peripheral blood mononuclear cells (PBMCs) isolation and macrophages differentiation

Peripheral blood mononuclear cells were isolated from buffy coats by Ficoll‐Hypaque density‐gradient centrifugation. The cells were gently incubated in red blood cell lysis buffer (Sigma‐Aldrich) for 2 minutes and washed with PBS (pH 7.4). Subsequently, PBMCs suspended in serum‐free RPMI 1640 medium (Gibco) supplemented with 1% penicillin/streptomycin were seeded in a six‐well plate for 1 hour in a humidified incubator containing 5% CO_2_ at 37°C to allow monocyte adhesion. Nonadherent cells were removed and the adherent monocytes were further incubated in RPMI 1640 medium supplemented with 10% (v/v) heat‐inactivated human serum and 1% penicillin/streptomycin for 7 days and media replacement every 3 days to obtain matured macrophages.^17^

### 2.4 Exosome isolation and labelling

Exosomes were isolated from the cell culture media with Total Exosome Isolation Reagent according to the manufacturer's instructions (Thermo Scientific, #4478359). The concentration of exosomal proteins was quantified with a BCA Protein Assay Kit (Thermo Scientific). The purified exosomes were then labelled with the green fluorescent linker PKH67 (Sigma) according to the manufacturer's guidelines.

### 2.5 Transmission electron microscopy

Exosomes to be examined by transmission electron microscopy (TEM) were isolated and loaded on to a carbon‐coated electron microscopy grid. The samples were fixed with 2% glutaraldehyde and 2% paraformaldehyde in 0.1 mol/L sodium cacodylate buffer at pH 7.3 for 3 hours at room temperature. After air drying, samples were mounted on specimen stubs and visualized using a JEOL JEM‐1010 transmission electron microscope (JEOL Ltd., Tokyo, Japan).

### 2.6 EdU staining

DNA synthesis was analysed using a Cell‐Light EdU Apollo488 In Vitro Imaging Kit (RiboBio Co., Ltd, Guangzhou, China) per the manufacturer's instructions.

### 2.7 Colony formation assay

A total of 200 cells were seeded in 6‐well plates and treated with macrophage supernatant as described previously.^18^ The cells were then incubated for approximately 12 days until most of the colonies contained more than 50 cells. The colonies were then fixed with methanol and dyed with Giemsa solution. Clone formation efficiency was calculated as the (number of colonies/number of cells inoculated) × 100%.

### 2.8 Scratch assay

A scratch assay was performed to assess cell migration in vitro. First, cells cultured in macrophage supernatant were seeded in 6‐well plates until a confluent monolayer was formed. Then, upon confluence, cells were scratched with a 10 μL sterile pipette tip. Pictures were then taken of the scratch at different time‐points under the microscope. The cell migration rate was calculated as (width at 0 hours–width at different time‐points)/width at 0 hours.

### 2.9 Cell invasion assay

Cell invasion was tested using BD BioCoat Matrigel Invasion Chambers (BD Biosciences, San Jose, CA, USA). Briefly, cells were seeded in the upper compartment of a Transwell Boyden Chamber (6.5 mm, Costar, Cambridge, MA, USA) with a polycarbonate membrane (8 mm pore size) on the bottom. This compartment contained macrophage supernatant, while the lower chamber was filled with media supplemented with 10% FBS. After 24 hours, the filter was washed, fixed with methanol and stained with crystal violet. Cells on the lower surface of the filter were analysed using Image J (Wayne Rasband, National Institutes of Health, Bethesda, MD, USA).

### 2.10 Western blotting

Exosomes or cells were lysed in RIPA buffer containing protease inhibitors (Roche Diagnostics, Mannheim, Germany). A total of 15 μg of exosomes were separated by SDS‐PAGE and transferred to PVDF membranes (Millipore, Bedford, MA, USA). The membranes were then incubated with antibodies against cluster of differentiation CD63 (Abcam, Cambridge, MA, USA), CD81 (Thermo Scientific, cat#10630D), tubulin (Cell Signaling Technology, CST, Beverly, MA, USA, cat#86298), E‐cadherin (CST, cat#3195), snail (CST, cat#3879), vimentin (CST, cat#5741), serine/threonine‐protein kinase AKT (CST, cat#4691), p‐AKT (CST, cat#4060), extracellular signal‐related kinase (Erk; CST, cat#4695), p‐Erk (CST, cat#4695), MET (CST, cat#8198), or p‐MET (CST, cat#3077).

### 2.11 Immunofluorescence

A total of two samples were collected from GC patients with *H. pylori* infection or patients with *H. pylori*‐associated chronic gastritis at the Second Affiliated Hospital of Nanjing Medical University. Formalin‐fixed and paraffin‐embedded samples were cut into 5 μmol/L sections, which were then processed for immunofluorescence. Primary antibodies used for immunoblotting were polyclonal rabbit anti‐human‐p‐cMet (Cell Signaling, cat#3077) and mouse anti‐human CD206 (Thermo Scientific, cat#53‐2069‐41). Tissues were then stained with anti‐rabbit Alexa Fluor^®^ 568 conjugated secondary antibody (Thermo Scientific, cat#A10042) at room temperature for 2 hours as well as 4′, 6‐diamidino‐2‐phenylindole (DAPI) to stain the nucleus. This study was approved by the Ethics Committee of the Second Affiliated Hospital of Nanjing Medical University.

### 2.12 RNA isolation and qPCR

Total RNA was isolated from cells or mouse tissues using Trizol reagent (Invitrogen), following the manufacturer's instructions. The RNA was then analysed using real‐time qPCR with SYBR Green PCR Master mix (Roche Applied Science, Mannheim, Germany) on a StepOnePlus™ Real‐Time PCR System (Applied Biosystems, Foster City, CA, USA). The relative gene expression was normalized to β‐actin. Specific primer sets used for this assay included MET (forward: TGCAC AGTTG GTCCT GCCAT GA, reverse: CAGCC ATAGG ACCGT ATTTC GG); Rab27b (forward: TGGCA ACAAG GCAGA CCTAC CA, reverse: CTCCA CATTC TGTCC AGTTG CTG); IL‐6 (forward: TACAT CCTCG ACGCA TCTC, reverse: AGCTC TGGCT GTTCC TCAC); IL‐1β (forward: AAACA GATGA AGTGC TCCTT CCAGG, reverse: AAACA GATGA AGTGC TCCTT CCAGG); tumour necrosis factor (TNF)‐α (forward: CCAGG CATCA GATCA TCTTC, reverse: GGATG TTCGT CCTCC TCACA G); vascular endothelial growth factor (VEGF) (forward: GAGGG CAGAA TCATC ACGAA, reverse: GGGAA CGCTC CAGGA CTTAT); and β‐actin (forward: CACGA AACTC CTTCA ACTCC, reverse: CATAC TCCTG CTTGC TGATC).

### 2.13 Cytokine secretion measurement

Secreted human IL‐1β and IL‐6 in culture supernatants were quantified using ELISA kits (Thermo Scientific) according to the manufacturer's instructions. LY294002 (phosphatidylinositol 3‐kinase, PI3K inhibitor) and U0126 (Erk inhibitor) were obtained from Beyotime Biotechnology (Shanghai, China). Experiments were performed in triplicate.

### 2.14 Xenograft model

Nude mice (male, 6 weeks of age) were obtained from the Model Animal Research Center of Nanjing University (Nanjing, Jiangsu, China). MGC‐803 cells were incubated in the supernatant of THP‐1‐derived macrophages, which were stimulated with PBS, MET^+^ exosomes, MET^−^ exosomes, MET^−^ exosomes + IL‐1β (PeproTech, Rocky Hill, NJ, USA, #AF‐200‐01B, 1 ng/mL), or MET^+^ exosomes + IL‐1β neutralizing antibody (Abcam, #ab9722, 3 μg/mL). Then, subcutaneous xenografts were created in the flank regions of the mice (n = 5 mice per group) via injection of 5 × 10^6^ MGC‐803 cells in macrophage supernatant as described above. Tumours were weighed and measured every 3 days. Tumour volume was calculated as width × length × (width + length)/2. The mice were killed 28 days after injection, and the tumours were removed. All of the animal studies performed were approved by the Nanjing Medical University Ethics Review Board.

### 2.15 MET silencing

Lipofectamine 3000 (Invitrogen) was used to stably transfect short hairpin RNA (shRNA) plasmids into AGS cells according to the manufacturer's instructions. For transient MET silencing, the following target sequence was used: 5′‐CCGGC ATCAG AACCA GAGGC TTGGT CTCGA GACCA AGCCT CTGGT TCTGA TGTTT TTG‐3′. AGS cells transfected with either MET shRNA (shMET) or control shRNA (shCtrl) were then co‐cultured with *H. pylori* for 12 hours. The exosomes were isolated from the supernatant of the shRNA‐treated AGS cells to stimulate macrophages for 48 hours. Finally, the supernatant from the stimulated macrophages was used to treat GC cells. We then evaluated the proliferation, migration and invasion of these cells.

### 2.16 Statistical analysis

All of the results reported here are representative of at least three independent experiments. Statistical evaluations were made using Student's *t*‐tests (two‐tailed). The data are presented as the means ± standard deviation (SD). *P*‐values < 0.05 were considered statistically significant.

## 3 RESULTS

### 3.1 MET is expressed in exosomes derived from *H. pylori*‐infected AGS cells

**Figure 1:**
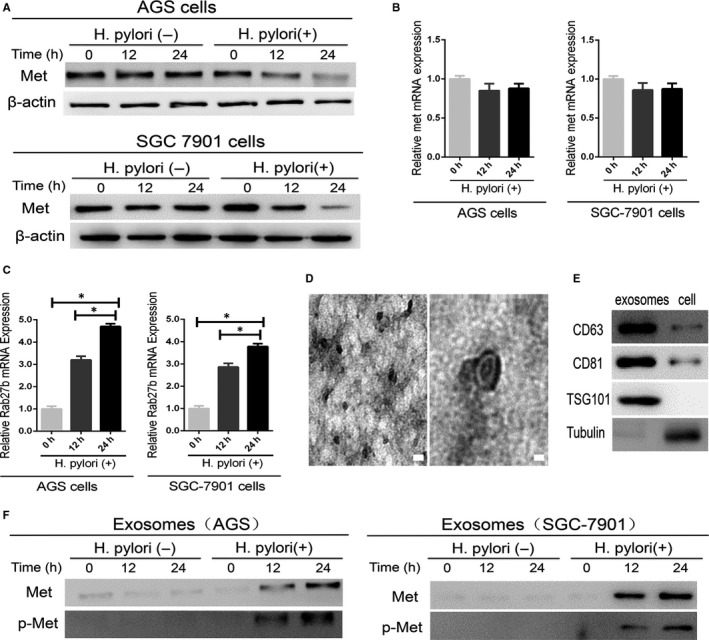
Mesenchymal‐epithelial transition factor (MET) expression in AGS cells and cell‐derived exosomes. A, Western blotting analysis was used to detect MET protein expression in AGS cells with and without *Helicobacter pylori* infection for the indicated times. B, qPCR analysis of MET mRNA levels in AGS cells infected with *H. pylori* for 0, 12 and 24 h. C, The relative expression of Rab27b mRNA was analysed by qPCR in AGS cells infected with *H. pylori* for 0, 12 and 24 h. *P < 0.05. D, Transmission electron microscopy image of exosomes derived from the AGS cells. Scale bars represent 100 nm. E, Western blotting analysis showing the presence of CD63 and CD81 and the absence of tubulin in AGS cell‐derived exosomes. F, MET and p‐MET expression in exosomes isolated from AGS cells with and without *H. pylori* infection was analysed by Western blotting analysis

As MET activation has been observed in *H. pylori*‐induced gastric tumorigenesis,^19^ we investigated the regulatory role of *H. pylori* infection on MET expression. Our Western blot analysis showed that *H. pylori* infection significantly reduced MET protein abundance in a time‐dependent manner (Figure 1A), whereas *MET* mRNA levels were unchanged (Figure 1B). We also observed a significant increase in Rab27b mRNA in AGS cells co‐cultured with *H. pylori* (Figure 1C). As Rab27b plays an important role in exosomes biogenesis,^20^ the exosomes were then isolated from the conditioned media of AGS cells infected with *H. pylori* for 24 hours. According to our TEM analysis, the purified exosomes appeared to be rounded particles ranging from 40 to 150 nm in diameter (Figure 1D). We further confirmed the presence of CD63, CD81 and TSG101, three specific exosome markers,^21^ and the absence of tubulin, in these AGS cell‐derived exosomes (Figure 1E). Notably, *H. pylori* infection induced a time‐dependent increase in MET expression in the exosomes. In contrast, MET protein was barely detectable in exosomes from uninfected AGS cells (Figure 1F). Phosphorylation at Tyr1234/1235 in the MET kinase domain is critical for kinase activation.^6^ Furthermore, our data showed that active p‐MET was also present in the exosomes from *H. pylori*‐infected AGS cells (Figure 1F). These results indicated that activated MET could be incorporated into exosomes during GC upon *H. pylori* infection.

### 3.2 Exosomes transferred MET into macrophages

**Figure 2:**
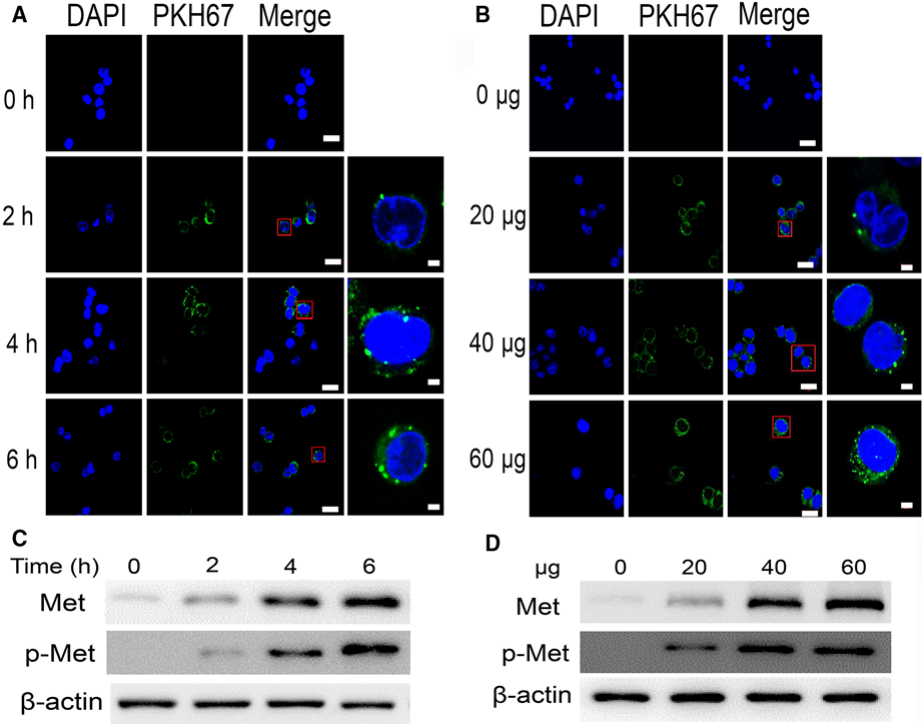
Exosomal mesenchymal‐epithelial transition factor (MET) is delivered to macrophages. A, Confocal microscopy image showing the internalization of 40 μg of PKH67‐labelled exosomes in macrophages at different time‐points. Scale bars represent 20 μm. B, Confocal microscopy image showing the internalization of 0, 20, 40, or 60 μg of exosomes into macrophages after 6 h. Magnification: 630×, scale bars represent 20 μm. Magnified views were shown on the right. Scale bars represent 2 μm. C,D, Graphs showing the time‐ and concentration‐dependent nature of exosome‐mediated MET expression in macrophages

Once secreted, exosomes deliver biological information to neighbouring or distant cells, thus modulating communication between tumour cells and the surrounding microenvironment.^22^ To examine the transfer of exosomal MET to recipient cells, macrophages were incubated with PKH67‐labelled exosomes derived from *H. pylori*‐infected AGS cells. These exosomes were found to enter macrophages in a time‐dependent (Figure 2A) and concentration‐dependent manner (Figure 2B), as highlighted by the green fluorescence in our confocal microscopy images. To further confirm exosomal ME internalization by macrophages, we also evaluated MET and p‐MET protein levels in macrophages. Before exosome treatment, MET was largely absent in the macrophages. However, after treatment with exosomes derived from *H. pylori*‐infected AGS cells, MET and p‐MET protein expression in the macrophages was increased in a time‐dependent and concentration‐dependent manner (Figure 2C,D). Taken together, these results clearly demonstrated that activated MET is transferred to and internalized by macrophages via exosomes.

### 3.3 Activated MET is preferentially expressed by tumour‐infiltrating macrophages

**Figure 3:**
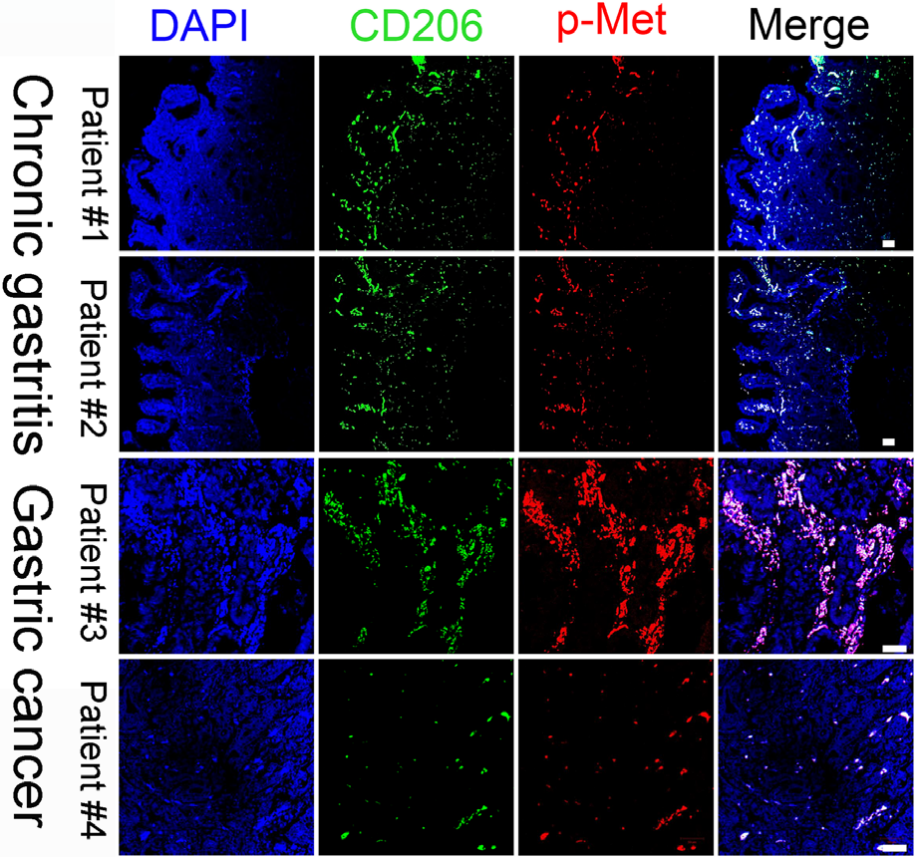
Activated mesenchymal‐epithelial transition factor (MET) is preferentially expressed in infiltrating macrophages from the patients with *Helicobacter pylori*‐positive chronic gastritis or gastric cancer. Representative images of tissues stained with the macrophage marker CD206 (green) and p‐MET (red). Scale bars represent 50 μm

We further evaluated the activated MET expression in macrophages present in human GC tissue positive for *H. pylori*. Interestingly, using the macrophage marker CD206,^23^ we found that macrophages were predominantly enriched in the GC tumour area. Furthermore, activated MET was highly expressed in these macrophages (Figure 3), supporting our theory that *H. pylori* infection modulates MET transfer to macrophages. In addition, immunofluorescence analysis shows that the number of CD206^+^ macrophages co‐expressing p‐MET was less in the tissues from the patients with *H.pylori*‐associated chronic gastritis than that in the patients with GC (Figure 3), indicating a potential role of MET in maintaining the tumorigenic function of the infiltrating macrophages.

### 3.4 Exosomal MET mediated the pro‐tumorigenic effects of macrophages

**Figure 4:**
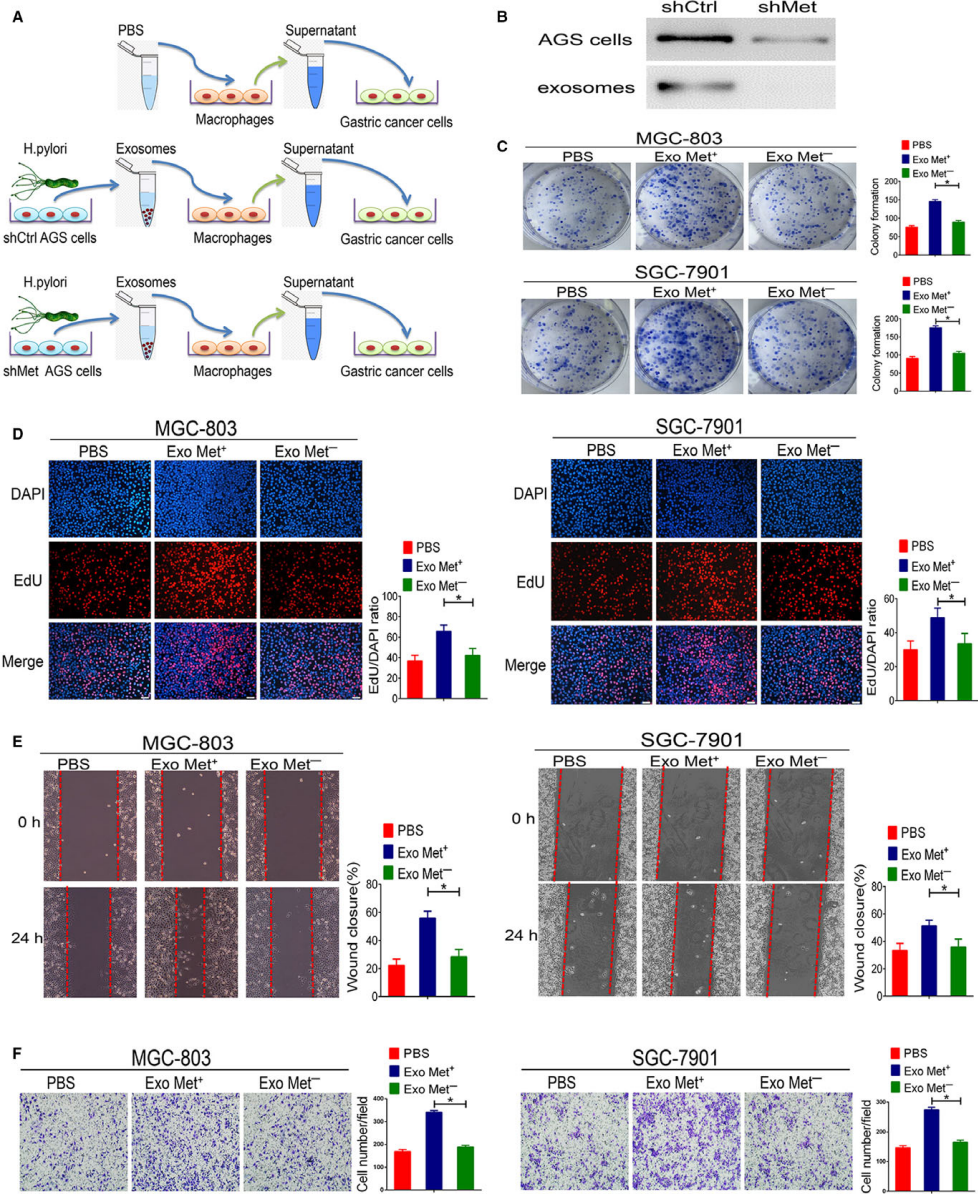
Exosomal mesenchymal‐epithelial transition factor (MET) mediates pro‐tumorigenic effects in macrophages. A, Schematic description of the experimental design. B, Suppression efficiency of MET shRNA in AGS cells or AGS cell‐derived exosomes was detected by Western blotting. C, Colony formation was evaluated based on GC cell proliferation. D, Representative images and quantification of DNA synthesis (EdU assay) in MGC‐803 and SGC‐7901 cells co‐cultured with supernatant from macrophages stimulated with PBS, MET^+^ exosomes, or MET^−^ exosomes. Scale bars represent 50 μm. E, Cell migration was assessed using a wound‐healing assay. F, A transwell assay was performed to assess GC cell invasion. Data represent at least three experiments performed in triplicate. *P < 0.05

To confirm that MET released from exosomes affects macrophages, we used shRNA to silence MET expression in AGS cells (please see Figure 4A for a schematic of this experiment). Notably, MET protein expression was dramatically reduced in *H. pylori* ‐infected AGS cells transfected with MET‐specific shRNA. MET was also absent from the exosomes isolated from MET‐deficient AGS cells infected with *H. pylori* (Figure 4B).

We next investigated whether the administration of MET^+^ exosomes would be sufficient to alter the biological effects of macrophages on GC cells. The results of our clonogenic cell survival assay demonstrate a significant increase in relative colony number in the macrophages pretreated with MET^+^ exosomes, whereas pretreatment with PBS and MET^−^ exosomes had a much weaker effect (Figure 4C). Preincubation of macrophages with MET^+^ exosomes also resulted in a greater increase in MGC‐803 and SGC‐7901 cell proliferation compared to that observed following macrophage preincubation with PBS or MET^−^ exosomes (Figure 4D). Consistent with these results, we found higher levels of GC cell migration after incubation with supernatant from macrophages treated with MET^+^ exosomes than in cells incubated with supernatant from macrophages treated with PBS or MET^−^ exosomes (Figure 4E). MGC‐803 and SGC‐7901 cell invasion were also substantially increased after being incubated with supernatant from macrophages treated with MET^+^ exosomes, whereas the supernatant from macrophages treated with PBS or MET^−^ exosomes did not have an effect (Figure 4F). These findings indicate that MET is required for exosome‐mediated activation of macrophages. Further, exosomal MET appears to be a crucial paracrine factor in mediating the pro‐tumour effects of macrophages, thus promoting GC development.

### 3.5 Exosomal MET increased the levels of IL‐1β secreted from macrophages via the Akt and MAPK pathway

**Figure 5:**
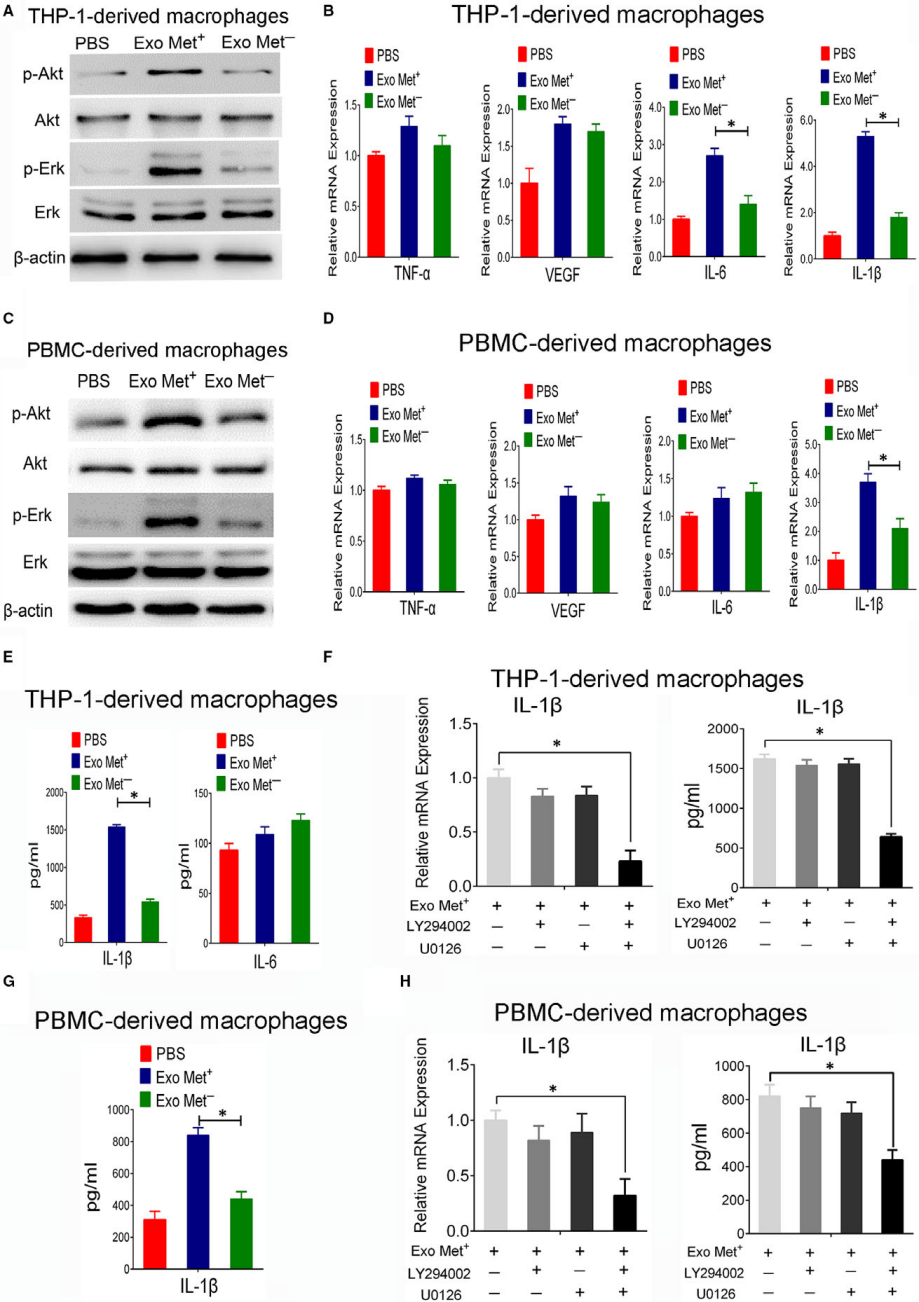
The effects of exosome‐delivered mesenchymal‐epithelial transition factor (MET) on macrophages. A,C, Western blot analysis of p‐Akt (Ser473) and p‐Erk (Thr202/Tyr204) protein expression in THP‐1‐ or PBMC‐derived macrophages stimulated with PBS, exosomes derived from AGS cells transfected with MET shRNA, or exosomes derived from AGS cells transfected with control shRNA following *Helicobacter pylori* infection. B,D, qPCR for TNF‐α, VEGF, IL‐6 and IL‐1β gene expression in THP‐1‐ (B) or PBMC‐derived macrophage (D) treated with PBS, MET^+^ exosomes, or MET^−^ exosomes. E,G, ELISA to detect the protein levels of secreted IL‐1β or IL‐6 from THP‐1 or PBMC‐derived macrophages treated with PBS, MET^+^ exosomes, or MET^−^ exosomes. F,H, qPCR and ELISA analysis for IL‐1β expression in THP‐1‐ (F) or PBMC‐derived macrophage (H) treated with LY294002 (20 μmol/L), U0126 (20 μmol/L) or a combination of both following MET^+^ exosomes stimulation. Data represent at least three experiments performed in triplicate. *P < 0.05

As the supernatant of macrophages incubated with exosomal MET appears to have a significant pro‐tumorigenic effect during GC pathogenesis, it was essential to evaluate the contents of the supernatant. Using Western blotting, we detected significantly increased activated Akt and Erk expression, two classical signalling transducers,^24^ in THP‐1‐derived macrophage and PBMC‐derived macrophages treated with MET^+^ exosomes (Figure 5A,C). To investigate the expression of classical proinflammatory factors in the supernatant from exosome‐treated macrophages, we performed qPCR to detect the mRNA levels of TNF‐α, VEGF, IL‐6 and IL‐1β.^24^, ^25^ Of these genes, IL‐6 and IL‐1β were significantly increased in THP‐1‐derived macrophages treated with MET^+^ exosomes compared to macrophages treated with PBS (Figure 5B), whereas only the mRNA level of IL‐1β was observed dramatically enhanced in PBMC‐derived macrophages treated by MET^+^ exosomes (Figure 5D). To evaluate protein secretion into the supernatant, an ELISA was conducted and the results show that IL‐1β protein expression was greatly elevated in the supernatant of THP‐1‐ and PBMC‐derived macrophages treated with MET^+^ exosomes (Figure 5E,G). Next, we seek to investigate whether the increase levels of IL‐1β from macrophages stimulated by MET^+^ exosomes was dependent on PI3K‐Akt or mitogen‐activated protein kinase (MAPK) pathway. As shown in Figure 5F,H, single treatment with LY294002 (PI3K‐Akt inhibitor) or U0126 (Erk inhibitor) did not cause significant changes on the mRNA and protein levels of IL‐1β induced by MET^+^ exosomes. However, the combination of LY294002 and U0126 successfully abolished MET^+^ exosomes‐mediated elevation of IL‐1β levels from THP‐1 and PBMCs‐derived macrophages (Figure 5F,H), which suggest that MET^+^ exosomes stimulated IL‐1β secretion from macrophages via the Akt and MAPK pathway.

### 3.6 Exosomal MET stimulates macrophages to facilitate tumour growth in vivo

**Figure 6:**
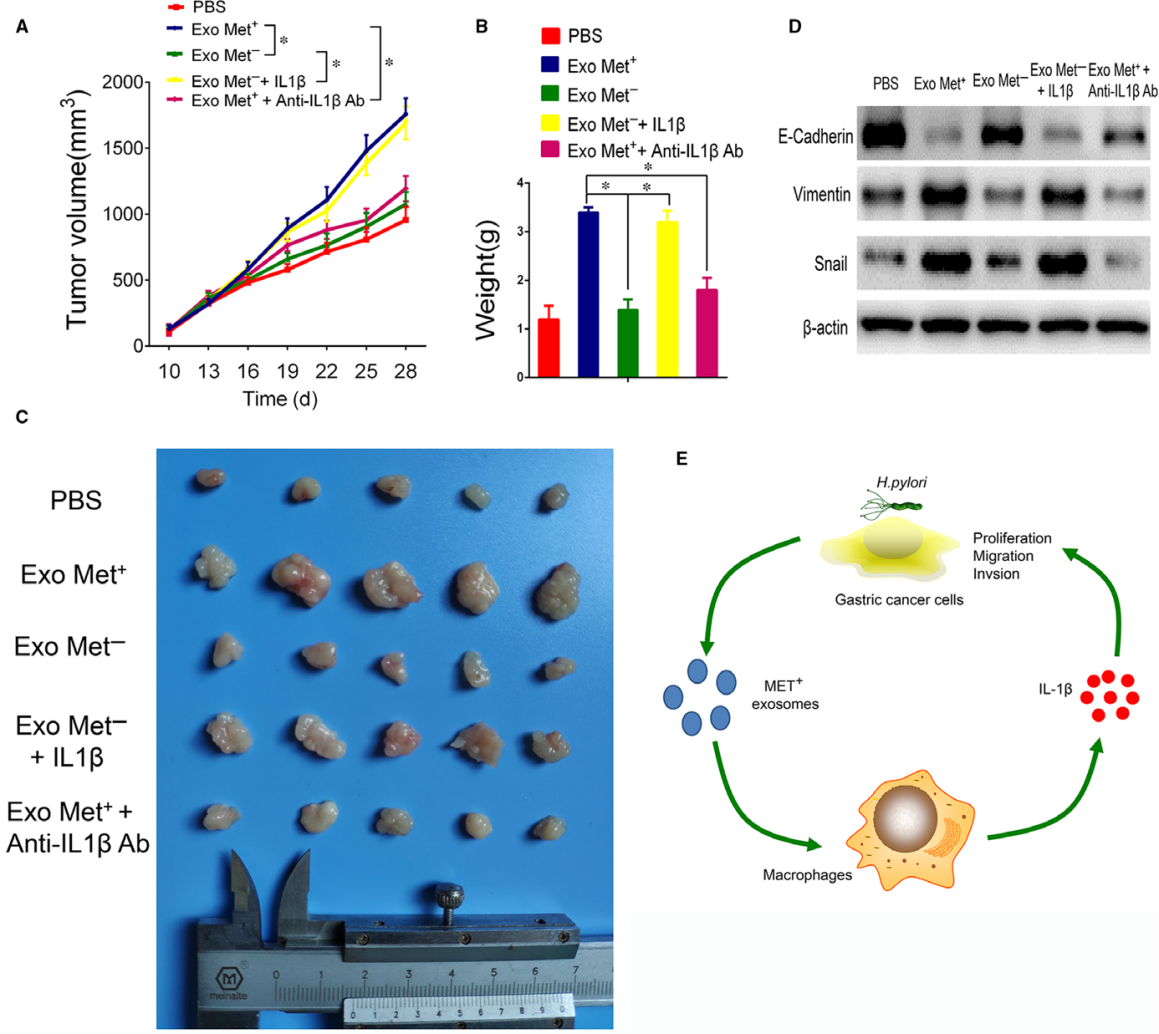
Exosome‐delivered mesenchymal‐epithelial transition factor (MET) stimulates macrophages to facilitate tumour growth in vivo. A, The effects of the supernatant from macrophage treated with PBS, MET^+^ exosomes, MET^−^ exosomes, MET^−^ exosomes + recombinant IL‐1β protein, MET^+^ exosomes + IL‐1β neutralizing antibody on tumour growth in a xenograft model. Tumour volume in the xenograft models was measured every 3 d after a 10 d inoculation period. B,C, Final tumour weights were determined and photographed. *P < 0.05, (n = 5). D, Western blotting analysis of E‐cadherin, vimentin and snail protein expression in GC cells. E, Model of the mechanism proposed in this study for exosomal MET in the tumour microenvironment following *Helicobacter pylori* infection. Our data indicate that exosomal MET stimulates tumour‐associated macrophages to release IL1‐β, which, in turn, promotes GC cells proliferation, invasion and migration

Using a xenograft model, we next investigated the role of exosomal MET in tumour growth in vivo. Treatment of GC cells with supernatant from macrophages incubated with MET^+^ exosomes caused a substantial increase in volume and weight of the resulting xenograft tumours compared to tumours formed from GC cells treated with supernatant from macrophages incubated with PBS or MET^−^ exosomes (Figure 6A,B). No significant changes in the tumour growth were observed between the MET^−^ exosome and PBS group. Ablation of IL‐1β by the neutralizing antibody withdrawn the malignant phenotypes. Concurrently, the addition of recombinant IL‐1β protein to the GC cells culture appeared to promote tumour progression in the MET^−^ exosomes group (Figure 6A‐C). Activation of an epithelial‐to‐mesenchymal transition (EMT) programme has been proposed as the critical mechanism for broadly regulating invasion and metastasis by epithelial cancer cells.^26^ EMT‐inducing transcription factors, such as snail, facilitate E‐cadherin loss, acquisition of a mesenchymal phenotype and expression of mesenchymal markers such as vimentin.^27^ As shown in Figure 6D, the supernatant from the macrophages treated with MET^+^ exosomes reduced the expression levels of E‐cadherin, while markedly increased the protein levels of vimentin and snail in GC cells. The incorporation of IL‐1β protein in MET^−^ exosomes treatment also caused similar effect as MET^+^ exosomes, while the addition of IL‐1β neutralizing antibody in MET^+^ exosomes treatment restored the expression of E‐cadherin (Figure 6D). Collectively, these data indicated that exosomal MET promoted IL‐1β secretion from macrophages leading to gastric cancer progression though EMT.

## 4 DISCUSSION

In this study, we investigated the exosome‐mediated transfer of MET between *H. pylori*‐infected GC cells and macrophage. Our results indicate that after *H. pylori* infection, GC cells secreted and transferred MET^+^ exosomes to the surrounding immune cells, which in turn, promoted GC tumorigenesis. This is likely related to the exosome‐mediated stimulation of proinflammatory cytokine IL‐1β secretion from the macrophages and activation of the Akt and MAPK pathways. To our knowledge, this is the first study to show that *H. pylori* infection induces exosome‐mediated transfer of MET into recipient cells, resulting in a feedback loop leading to GC pathogenesis.

To date, several mechanisms for negative regulation of MET expression have also been established in *H. pylori*‐infected GC cells. It is important to note that exosomal MET secreted from *H. pylori*‐infected GC cells exists stably in its phosphorylated form. This is a critical finding as MET phosphorylation is essential for its function. Typically, the intracellular tyrosine kinase catalytic domain induces receptor activation and auto‐phosphorylation at Tyr1234 and Tyr1235, which are known docking sites for downstream signal transduction molecules.^5^ Our finding suggested that exosomal MET secreted from *H. pylori*‐infected gastric cancer cells existed stably in the tyrosine‐phosphorylated form.

Gastric cancer contains abundant tumour‐supportive macrophages to promote malignant progression.^28^ Macrophages infiltration is correlated with poor prognosis of gastric cancer patients. Accumulating evidence has shown that tumours can interfere with the immune system via secreted exosomes. For example, tumour‐derived exosomes were observed to transfer activated EGFR to host macrophages, thereby suppressing innate antiviral immunity.^29^ In the present study, we clearly demonstrated that macrophages internalized exosomal MET at a high efficiency. In addition, the expression of MET was higher in tumour‐associated macrophages in the *H. pylori*‐infected human GC tissues. We, therefore, focused our attention on determining the effects of macrophages treated with exosomes containing activated MET on GC malignancy and tumour progression. Notably, macrophages treated with MET^+^ exosomes markedly promoted GC growth and invasion both in vivo and in vitro. In contrast, macrophages incubated with PBS or MET^−^ exosomes did not significantly alter GC progression. Therefore, our data suggest that exosomal MET may be a potential regulator of the pro‐tumorigenic effect of macrophages in GC pathogenesis.

To better understand the mechanisms underlying the observed exosomal MET‐mediated effects on macrophage function and, subsequently, GC progression, we focused on changes in inflammatory mediators known to play a role in the tumour microenvironment. Chronic inflammation is recognized as a tumour hallmark and is implicated in nearly all stages of tumorigenesis.^30^ In this study, we found that exosome‐treated macrophages preferentially secrete the proinflammatory cytokines IL‐1β and activated the Akt and MAPK pathways. IL‐1β plays a pivotal role in proliferation, migration and invasion in malignant tumours.^31^, ^32^ In fact, IL‐1β is suspected to be the primary factor determining why some individuals infected with *H. pylori* develop GC while others do not.^33^ While our data support this theory, the network regulating the expression and function of MET in tumour‐activated macrophages is complex and still requires significant exploration. Several studies have described that exosomes derived from gastric cancer cells are transferred or delivered to modulate biological function and signalling of recipient cells.^34^ Our findings are in agreement with these studies suggesting that exosomes have critical roles in the tumour microenvironments by enhancing communication in both gastric cancer cells and macrophages.

In conclusion, we have demonstrated that *H. pylori* infection establishes a feedback loop between macrophages and GC cells via exosome‐mediated transfer of activated MET. Our data also suggest that exosomal MET contributes to the uncontrolled activation of macrophages and downstream inflammation, including the secretion of IL‐1β. This mechanism may explain how *H. pylori* infection results in gastric tumorigenesis. As macrophage infiltration may contribute to poor prognosis in GC patients, targeting macrophages by blocking MET may synergize with conventional therapies to improve patient survival. While additional work is required to test this, the present study defines the role of exosomal MET in the activation of macrophages and highlights this mechanism as a potential drug target.

## CONFLICT OF INTEREST

The authors declare that they have no competing interests.

## AUTHOR'S CONTRIBUTIONS

YC, BG, YX, ZL and QY designed the experiments. YC, BG and YX performed the research. YC, BG, YX, XM, LC, XM, JP, CZ, TZ, CW, XL and HW analysed the data. YC and QY wrote the manuscript.
